# Comprehensive analysis of microarray expression profiles of circRNAs and lncRNAs with associated co-expression networks in human colorectal cancer

**DOI:** 10.1007/s10142-018-0641-9

**Published:** 2018-11-16

**Authors:** Yan Tian, Yu Xu, Huawei Wang, Ruo Shu, Liang Sun, Yujian Zeng, Fangyou Gong, Yi Lei, Kunhua Wang, Huayou Luo

**Affiliations:** 1 Department of Gastrointestinal and Hernia Surgery, The First Affiliated Hospital of Kunming Medical University, No. 295 Xichang Road, Kunming, 650032 China; 2 Yunnan Institute of Digestive Disease, The First Affiliated Hospital of Kunming Medical University, No. 295 Xichang Road, Kunming, 650032 China; 3 Kunming Engineering Technology Center of Digestive Disease, No. 295 Xichang Road, Kunming, 650032 China

**Keywords:** Colorectal cancer, circRNAs, lncRNAs, GO analysis, Pathway analysis, Co-expression analysis

## Abstract

Increasing data demonstrate that circular RNAs (circRNAs) and long non-coding RNAs (lncRNAs) play important roles in tumorigenesis. However, the mechanisms in colorectal cancer (CRC) remain unclear. Here, hundreds of significantly expressed circRNAs, and thousands of lncRNAs as well as mRNAs were identified. By qRT-PCR, one abnormal circRNA, lncRNA, and three mRNAs were verified in 24 pairs of tissues and blood samples, respectively. Then, by GO analysis, we found that the gene expression profile of linear counterparts of upregulated circRNAs in human CRC tissues preferred positive regulation of GTPase activity, cellular protein metabolic process, and protein binding, while that of downregulated circRNAs of CRC preferred positive regulation of cellular metabolic process, acetyl-CoA metabolic process, and protein kinase C activity. Moreover, Kyoto Encyclopedia of Genes and Genomes (KEGG) pathway analysis showed that p53 signaling pathway was an important pathway in upregulated protein-coding genes, whereas cyclic guanosine monophosphate-protein kinase G (cGMP–PKG) signaling pathway was the top enriched KEGG pathway for downregulated transcripts. Furthermore, lncRNA–mRNA co-expression analysis demonstrated that downregulated lncRNA uc001tma.3 was negatively with CDC45 and positively with ELOVL4, BVES, FLNA, and HSPB8, while upregulated lncRNA NR_110882 was positively with FZD2. In addition, lncRNA–transcription factor (TF) co-expression analysis showed that the most relevant TFs were forkhead box protein A1 (FOXA1), transcription initiation factor TFIID submint 7 (TAF7), and adenovirus early region 1A(E1A)-associated protein p300 (EP300). Our findings offer a fresh view on circRNAs and lncRNAs and provide the foundation for further study on the potential roles of circRNAs and lncRNAs in colorectal cancer.

**Electronic supplementary material:**

The online version of this article (10.1007/s10142-018-0641-9) contains supplementary material, which is available to authorized users.

## Background

Colorectal cancer (CRC), one of the most common causes of cancer-related deaths in the world, leads to 600,000 deaths each year worldwide (Sostres et al. 2014; Ung et al. 2014). And CRC is becoming more prevalent in China (Li et al. 2013). Although current detection methods, such as fecal occult blood testing and fiber-optic colonoscopy, have significantly improved outcomes for CRC patients, more than half of those at advanced stage of disease die of cancer recurrence after suffering radical resection (Edwards et al. 2014). Therefore, fresh biomarkers for a more exact prediction of colorectal cancer recurrence and cancer therapy are urgently needed.

It is well known that the ratio of transcribed human genome was approximately 5 to 10%. Among human transcripts, about 10–20% were the protein-coding RNAs, and the remaining 80–90% were non-protein-coding transcripts, namely non-coding RNAs (Bertone et al. 2004; Kapranov et al. 2007). Circular RNAs (circRNAs), a widespread form of non-coding RNA in mammalian cells, were formed by non-sequential back-splicing of pre-mRNA transcripts (Conn et al. 2015; Memczak et al. 2013). Other than linear RNAs that are terminated with 5′caps and 3′tails, circRNAs form covalently closed loop structures with neither 5′–3′ polarities nor polyadenylated tails (Chen and Yang 2015). CircRNAs modulate gene expression at the transcriptional or post-transcriptional level by interplaying with microRNAs (miRNAs) or other molecules (Hansen et al. 2013). Due to circRNAs interact with miRNAs to regulate their target genes, circRNAs could likely be engaged in diseases correlated with miRNAs (Qu et al. 2015). It has been reported that circRNAs could mediate cancer progression (Li et al. 2015b). In CRC, global reduction circRNA abundance and proliferation revealed a negative correlation (Bachmayr-Heyda et al. 2015). However, the research about the global functional roles of circRNAs in CRC was few reported.

Long non-coding RNA (lncRNA), more than 200 bp, is a type of non-coding RNA molecule that can modulate gene expression in transcriptional or post-transcriptional level (Cheetham et al. 2013; Guttman and Rinn 2012). According to their relative position in the genome to the protein-coding genes, lncRNAs can be divided into five categories: sense, antisense, intronic, intergenic, and bidirectional (Mercer et al. 2009). LncRNAs play in or *trans* (distally to modulate gene expression across multiple chromosomes) (Lee 2012). It has been reported that lncRNAs play significant roles in various physiological and pathophysiological processes (Di Gesualdo et al. 2014; Fatica and Bozzoni 2014). Moreover, increasing evidence indicated that many lncRNAs reveal significant roles in the regulation of CRC. Among these lncRNAs, the upregulation of HOTAIR, MALAT1, CCATA2, and the downregulation of LOC285194, UC.388, and LET have been implicated in promoting metastasis of CRC (Ye et al. 2015). However, to date, little is known about the comprehensive function analysis of lncRNAs in CRC.

Therefore, here, firstly, we investigated the differentially expressed profiles of circRNA, lncRNA, and mRNA in colorectal cancer using microarray to understand the molecular patterns of non-coding RNAs for colorectal cancer. Then, using qRT-PCR, we further verified the significant differentially expresses of typical circRNA, lncRNA, and mRNAs. Moreover, we not only performed for Gene Ontology and pathway analysis but also depicted general functional landscapes of coding-non-coding co-expression, nearby coding of lncRNA and TF-lncRNAs enriched with mRNA network for the first time with specific bioinformatics methods in colorectal cancer. In addition, our data revealed that there were several circRNAs and lncRNAs which could be regarded as diagnostic and therapeutic markers for CRC. Finally, by researching the co-expression networks of these differentially expressed lncRNAs and mRNA, we further estimated the functions of lncRNAs. We believe that our findings might clarify the new pathophysiological mechanism of CRC and offer novel targets for CRC.

## Material and methods

### Patients and samples

A total of 30 patients who underwent surgical resections at the First Affiliated Hospital of Kunming Medical University between January 2013 and December 2016 were used for our study. Before the operation, these patients were histologically confirmed to be colorectal cancer and received neither chemotherapy nor radiotherapy. Thirty pairs (grades I~IV) of colorectal cancer tissue (A) and adjacent normal tissue samples (N) were obtained, frozen in liquid nitrogen immediately after surgical resection, and stored at − 80 °C until use. Five milliliters of venous blood was sampled from 24 colorectal cancer patients (grades I~IV) prior to surgery and 24 healthy controls, respectively. Serum from blood sample was centrifuged for 10 min at 4000 rpm and then stored at − 80 °C until use. Six pairs (grade IV of colorectal cancer tissue (A) and adjacent normal tissue samples (N) was used for microarray assay), 24 pairs (grades I~IV) of colorectal cancer tissue (A) and adjacent normal tissue samples (N) and serum of 24 colorectal cancer patients (grades I~IV), and 24 healthy controls were used for detecting genes using qRT-PCR. Written informed consents were received from all the participants, and this study was approved by the ethics committee of the First Affiliated Hospital of Kunming Medical University.

### RNA extraction and quantification control

Based on the manufacture’s protocol, total RNA was extracted from colorectal adenocarcinoma tissues and adjacent non-tumor tissues, serum of 24 colorectal cancer patients (grades I~IV), and 24 healthy controls using Trizol reagent (Invitrogen, USA). RNA quantity and quality were tested by NanoDrop ND-1000 spectrophotometer (OD 260 nm, NanoDrop, Wilmington, De, USA). And RNA integrity was evaluated by standard denaturing agarose gel electrophoresis.

### Microarray assay

Six pairs of colorectal adenocarcinoma tissues and adjacent non-tumor tissues were used for microarray assay to detect differentially expressed circRNA, lncRNA, and mRNAs comparing tumor group and normal group. Based on the Agilent One-Color Microarray-Based Gene Expression Analysis protocol (Agilent Technology) with minor modifications, sample labeling and array hybridization were conducted. Briefly, mRNA was purified from total RNA, amplified, transcribed into fluorescent cRNA, and then hybridized into the Human circRNA microarray V2 (Arraystar) and Human lncRNA microarray V4.0 (Arraystar). Finally, the hybridized arrays were washed, fixed, and scanned using the Agilent Scanner G2505C.

### Data analysis

Acquired array images were analyzed by Agilent feature extraction software (version 11.0.1.1). The raw data were quantile normalized, and subsequent data analysis was performed with the R software limma package and GeneSpring GX v12.1 software package (Agilent Technologies). The significant differentially expressed circRNAs, lncRNAs, and mRNAs between tumor group and normal group was determined by *p* value and false discovery rate (FDR) filtering. Significant differentially expressed circRNAs, lncRNAs, and mRNAs were retained through screening fold change ≥ 2.0, *P* < 0.05, and FDR < 0.05. An overview of the characteristics of expression profiles based on values of all expressed circRNAs, lncRNAs, and mRNAs and significant differentially expressed circRNAs, lncRNAs, and mRNAs was generated by hierarchical clustering.

### qRT-PCR verification

Forty-eight pairs of CRC samples (including 24 pairs of colorectal cancer tissue (A) and adjacent normal tissue samples (N), and blood of 24 colorectal cancer patients (grades I~IV) and 24 healthy controls) were performed for qRT-PCR determination. After total RNAs were extracted, they were reverse transcribed to cDNA based on the protocol of manufacturer. Subsequently, qRT-PCR was performed in a total reaction volume of 10 μl, including 0.5 μl PCR Forward Primer (10 μM), 0.5 μl PCR Reverse Primer (10 μM), 2 μl cDNA, 5 μl 2× Master Mix, and 2 μl double distill water. The protocol was initiated at 95 °C for 10 min, then at 95 °C (10 s), and 60 °C (60 s) for a total 40 cycles. β-Actin was seen as a reference gene. Relative expression level was calculated using ΔCt method. *P* value < 0.05 was considered to be significant. The primers are below. hsa_circRNA_100085: F, 5′-CCTACCCCATCCCCTTATTC-3′; R, 5′-ACCGTGCTGTAGACTGCTGAG-3′. LncRNA AK021804: F, 5′-GGTTTCTCAACACCCCTTCTTC-3′; R, 5′-CCCCAGTTCCCCGCCTTAT-3′. IFNG: F, 5′-GAGTGTGGAGACCATCAAGGAAG-3′; R, 5′-GGCGACAGTTCAGCCATCAC-3′. ITGA5: F, 5′-ACCCAGACCCTGCTCATCCA-3′; R, 5′-TGTGAATCGGCGAGAGTTTGTC-3′. DES: F, 5′-AGGACCTGCTCAACGTGAAGAT-3′; R, 5′-CTTTGCTCAGGGCTGGTTTCT-3′. β-actin F, 5′-AGCACAGAGCCTCGCCTTTG-3′; R, 5′-CTTCTGACCCATGCCCACCA-3′.

### GO and pathway analysis

The Gene Ontology (GO) analysis was performed for constructing meaningful annotation of genes and gene products in any organism (http://www.geneontology.org). GO includes three domains: biological process (BP), cellular component (CC) and molecular function (MF). Fisher’s exact test was used to confirm whether the overlap between the differentially expressed (DE) list and the GO annotation list is greater than that expected by chance. The *P* value reveals the significance of the GO term enrichment in the DE genes. FDR stands for the false discovery rate. The lower the *P* value is, the more significant the GO term (a P value < 0.05 is adopted). Pathway analysis, a functional analysis, mapped genes to Kyoto Encyclopedia of Genes and Genomes (KEGG) pathways (http://www.genome.jp/kegg/). The *P* value (EASE score, Fisher *P* value or hypergeometric *P* value) shows the significance of the pathway correlated to the conditions. The lower the *P* value is, the more significant considered the pathway.

### Correlation and co-expression analysis

Through calculating the Pearson correlation coefficient (PCC) between coding genes and non-coding transcripts basing on their expression levels, the co-expression analysis was conducted. The absolute value of parameter PCC ≥ 0.90, *P* value < 0.01, and FDR < 0.01 was accepted and reserved for further analysis.

### *Trans* regulation predication

It has been defined that a *trans* regulator does not meet the criterion. And lncRNAs can modulate gene expressions by *trans* manner. The important differentially expressed lncRNAs whose expression levels were associated with that of mRNAs to *trans* prediction. For *trans* predication, we concentrated on the manner that lncRNAs execute their functions through transcription factors (TFs). Thus, those mRNAs co-expressed with lncRNAs significantly overlapped with the target genes of a given TF were enriched and the TF–lncRNA–mRNA networks were built.

#### Data availability

The dataset and materials presented in this investigation are available by request from the corresponding author.

## Results

### Differentially expressed circRNA, lncRNA and mRNA profiles in human colorectal cancer tissues

**Fig. 1:**
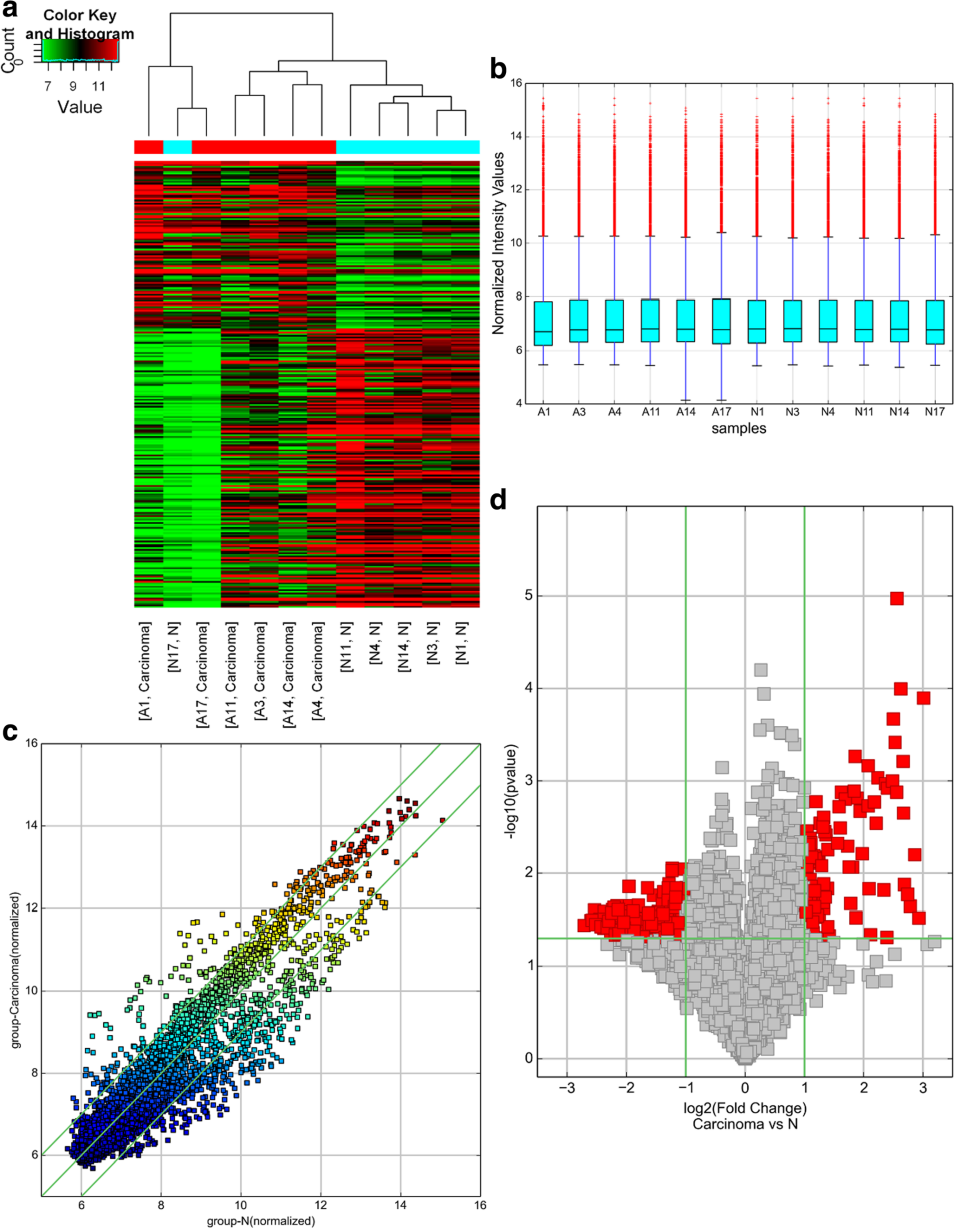
Differentially expressed circular RNA (circRNAs) in human CRC tissues (A) and adjacent normal tissues (N). **a** Hierarchical clustering of differentially expressed circRNAs. **b** The box plot shows the variations in circRNA expression. **c** The scatter plot explains the distribution of the data in circRNA profiles. The values of the *x*- and *y*-axes in the scatter plot were the averaged normalized signal values of the group (log_2_ scales). **d** The volcano plot illustrates the distribution of the data in circRNA profiles. The green lines in the scatter and volcano plots show the significant fold change (color figure online)

**Fig. 2:**
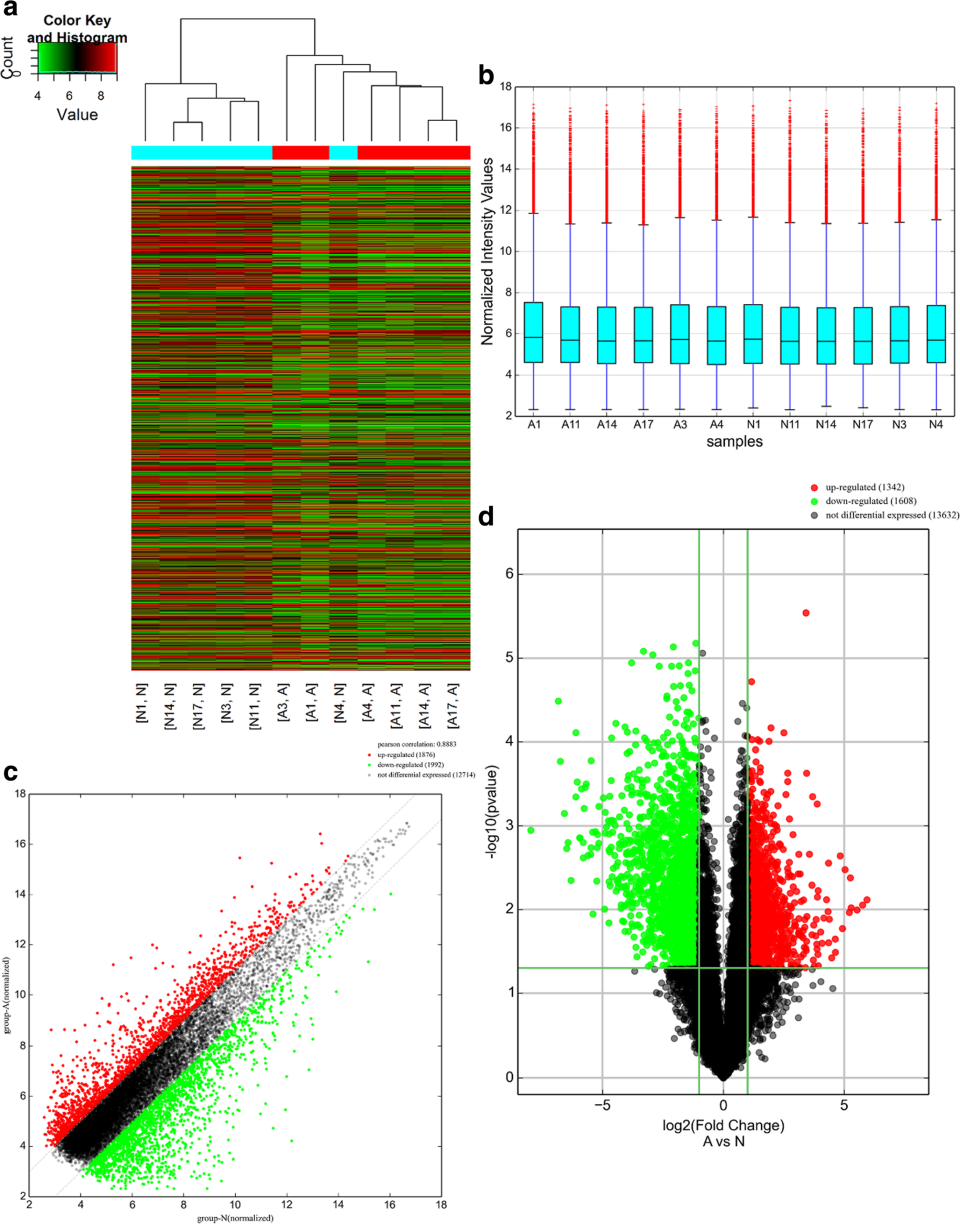
Differentially expressed long non-coding RNAs (lncRNAs) in human CRC tissues (A) and adjacent normal tissues (N). **a** Hierarchical clustering of differentially expressed lncRNAs. **b** The box plot shows the variations in circRNA expression. **c** The scatter plot explains the distribution of the data in lncRNA profiles. The values of the *x*- and *y*-axes in the scatter plot were the averaged normalized signal values of the group (log_2_ scales). **d** The volcano plot illustrates the distribution of the data in lncRNA profiles. Red, green, and black points in the scatter and volcano plots represent significant upregulated lncRNAs; significant downregulated lncRNAs and not differential expressed lncRNAs, respectively (color figure online)

**Fig. 3:**
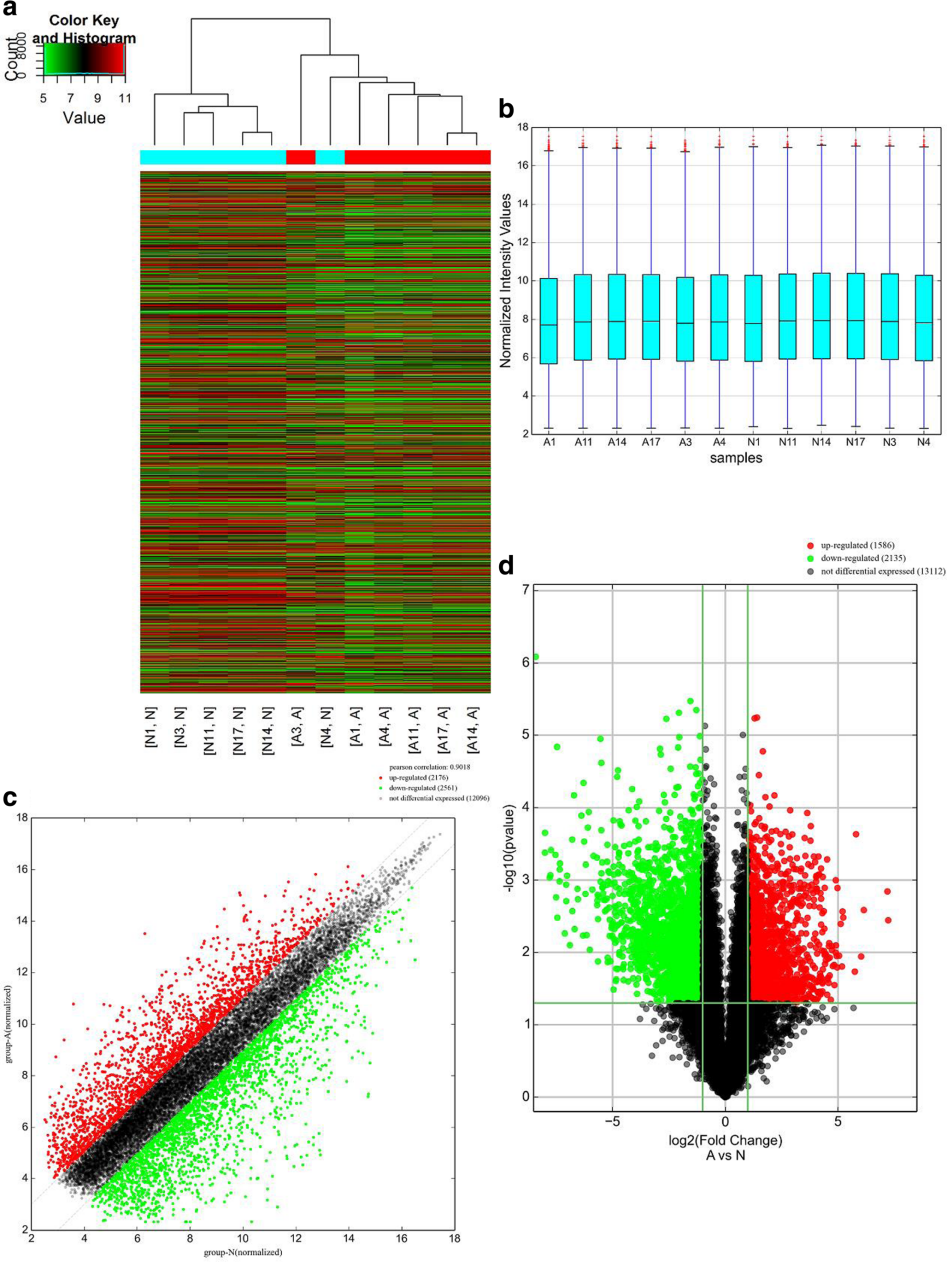
Differentially expressed mRNAs in human CRC tissues (A) and adjacent normal tissues (N). **a** Hierarchical clustering of differentially expressed mRNAs. **b** The box plot shows the variations in circRNA expression. **c** The scatter plot explains the distribution of the data in mRNA profiles. The values of the *x*- and *y*-axes in the scatter plot were the averaged normalized signal values of the group (log_2_ scales). **d** The volcano plot illustrate the distribution of the data in mRNA profiles. Red, green and black points in the scatter and volcano plots represent significant upregulated mRNAs, significant downregulated mRNAs and not differential expressed mRNAs, respectively (color figure online)

**Fig. 4:**
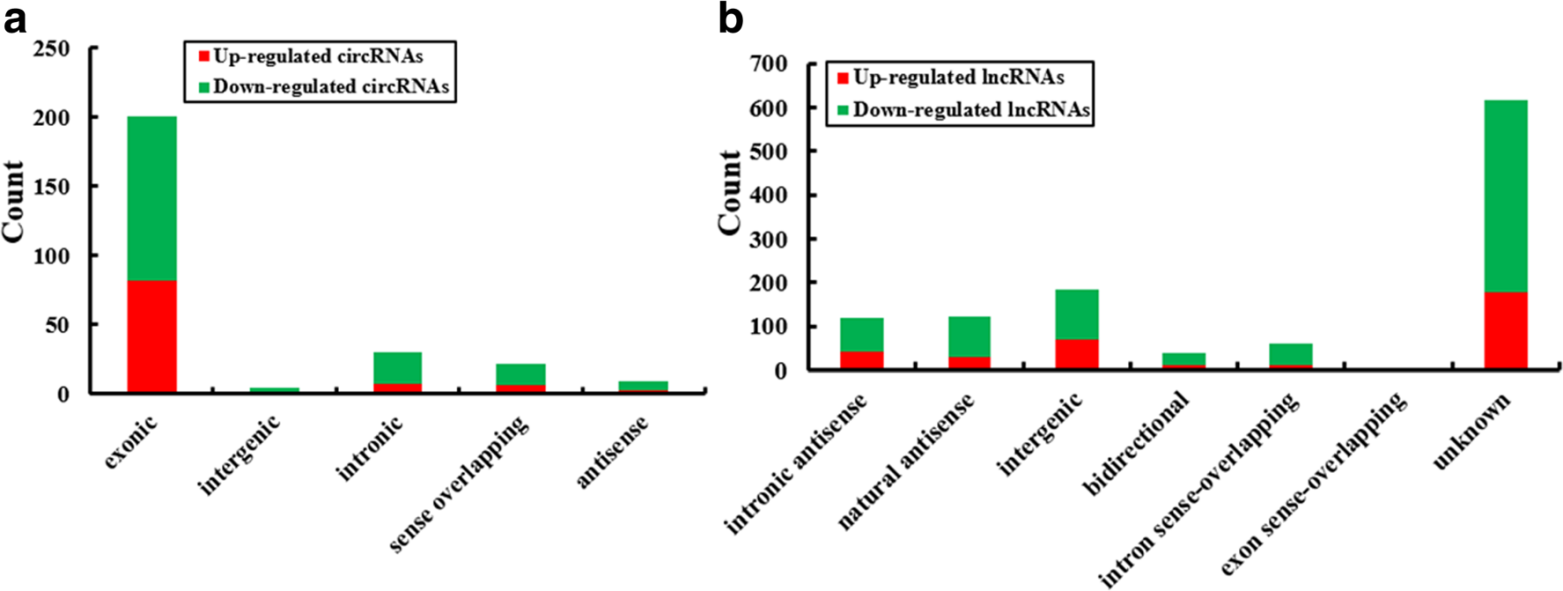
Classification of differentially expressed circRNAs and lncRNAs in CRC tissues. **a** Types and counts of differentially regulated circRNAs detected by microarray (fold change ≥ 2.0, *P* < 0.05 and FDR < 0.05). The circRNAs are classified into five types according to the relationship and genomic loci with their associated coding genes. **b** Types and counts of differentially regulated lncRNAs detected by microarray (fold change ≥ 2.0, *P* < 0.05 and FDR < 0.05). The lncRNAs are classified into six types according to the relationship and genomic loci with their associated coding genes

A genome-wide analysis was used to profile difference in circRNA, lncRNA, and mRNA expressions between human CRC tissues and adjacent normal tissues (Table S1, Table S2, and Table S3, respectively). A total of 265 circRNAs were found to be differentially expressed with fold change ≥ 2.0, *P* < 0.05 and FDR < 0.05 between human CRC tissues and adjacent normal tissues, among them, 99 upregulated and 166 downregulated (Fig. 1). Among the dysregulated circRNAs, based on their relation with protein-coding genes, the circRNAs were classified into five categories: 75.85% were exonic, 1.51% were intergenic, 11.32% were intronic, 7.92% were sense overlapping, and 3.4% were antisense (Fig. 4a). Total 1148 lncRNAs and 1553 mRNAs between human CRC tissues and adjacent normal tissues were with fold change ≥ 2.0, *P* < 0.05, and FDR < 0.05 (Figs. 2 and 3). Among them, 338 and 810 lncRNAs were upregulated and downregulated; meanwhile, 553 and 1000 mRNAs were upregulated and downregulated, respectively. Two hundred and forty-six lncRNAs revealed fold change ≥ 10, including 8 upregulated lncRNAs and 138 downregulated lncRNAs. T115989 (fold change ~ 33) was the most upregulated lncRNA. NR_003679 (fold change ~ 249) was the most downregulated lncRNA. Summarization of coding gene profile showed that 328 mRNAs displayed fold change ≥ 10 (up 65; down 263). CircRNA, lncRNA, and mRNA expression patterns among samples were different as shown by hierarchical clustering. These data indicated that the expressions of circRNAs, lncRNAs, and mRNAs in human CRC tissues are distinguished from those in corresponding non-tumor tissues (Figs. 1, 2, and 3). Among the dysregulated lncRNAs, basing on their relation with protein-coding genes, the lncRNAs were classified into six categories: 10.45% were intronic antisense, 10.54% were natural antisense, 16.11% were intergenic, 3.4% were bidirectional, 5.23% were intron sense-overlapping, and 0.44% were exon sense-overlapping (Fig. 4b).

### Verification of abnormal circRNA, lncRNA, and mRNAs

**Fig. 5:**
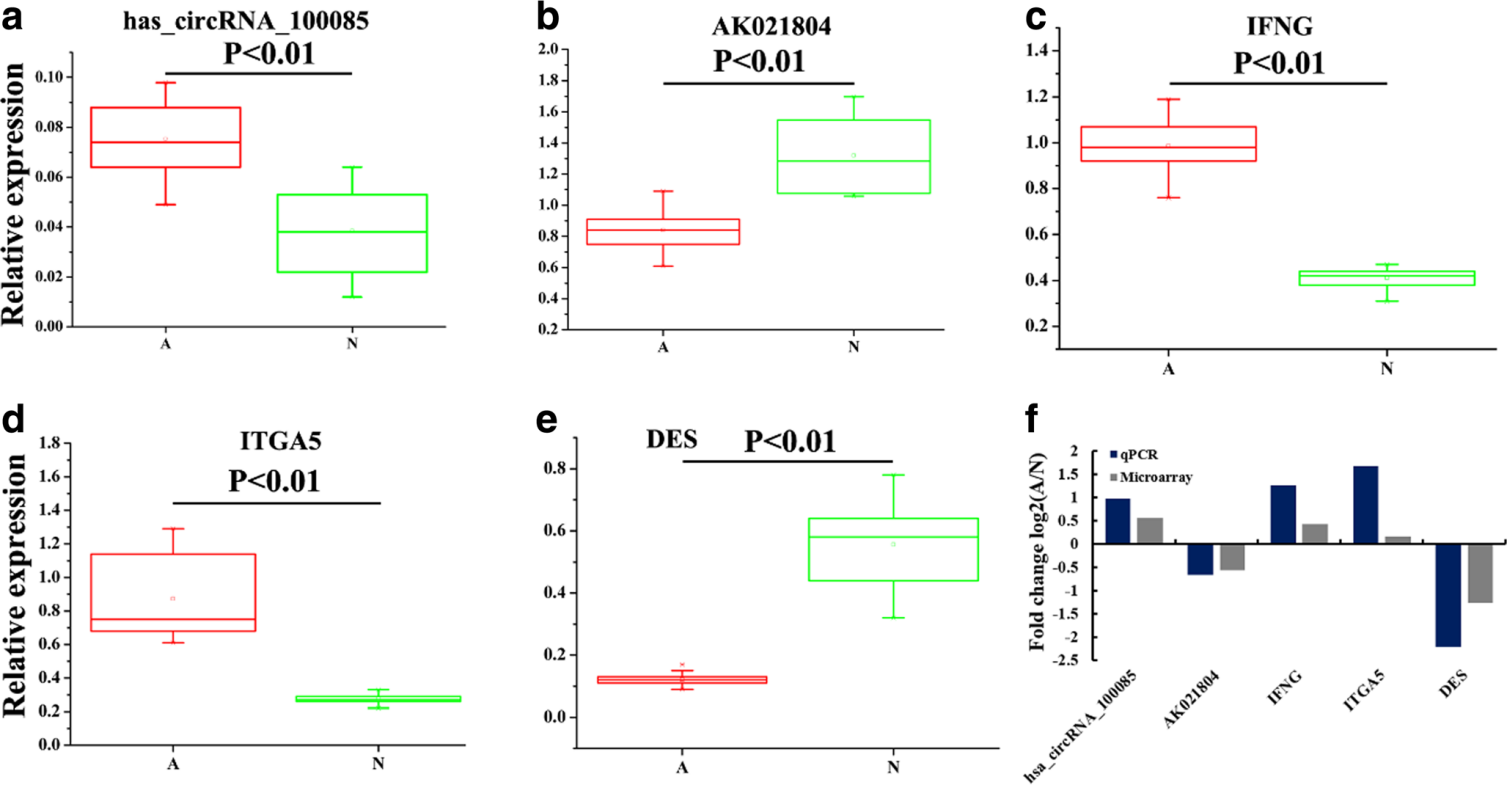
Verification for the expression of significant transcripts by qRT-PCR. **a–e** Relative expression levels of one circRNA, one lncRNA, and three mRNAs are shown comparing tumor tissues (A) and adjacent normal tissues (N). **f** A good correlation of qRT-PCR results and microarray data are shown through comparing such two results. The heights of the columns stand for the fold changes (log2 transformed) computed from the qRT-PCR and microarray data

By qRT-PCR, one circRNA, one lncRNA, and three mRNAs were selected for validating the microarray results in 24 colorectal cancer tissue (A) and adjacent normal tissue samples (N) and blood of 24 colorectal cancer patients (grades I~IV) and 24 healthy controls. For 24 pairs of tissues samples, qRT-PCR results showed that the expressions of circRNA_100085, interferon gamma (IFNG), and itergrin alpha-5 (ITGA5) were upregulated, while AK0218041 and DES were downregulated (Fig. 5a–e). These results are similar with the microarray assay (Fig. 5f). Therefore, the qRT-PCR results validated the veracity of microarray data. As shown in Table 1, circRNA_100085, AK02180, IFNG, ITGA5, and DES were significantly associated with tumor size and TNM. However, no significant correlations were found between the five genes and gender and age. For blood of 24 colorectal cancer patients (grades I~IV) and 24 healthy controls, their results show similar results of tissues (Fig. S1; Table S4).

**Table 1:** Clinical characteristic of 24 CRC patients and the expression of five genes in tissues

Characteristics	Case	has_circRNA_100085	*P*	AK021804	*P*	IFNG	*P*	ITGA5	*P*	DES	*P*
Gender			0.638		0.052		0.142		0.152		0.424
Male	14	1.02 ± 0.02		0.77 ± 0.10		1.46 ± 0.15		1.47 ± 0.22		0.73 ± 0.07	
Female	10	1.03 ± 0.02		0.67 ± 0.13		1.54 ± 0.11		1.63 ± 0.31		0.76 ± 0.07	
Age (years)			0.507		0.142		0.15		0.315		0.247
≥ 55	13	1.02 ± 0.02		0.77 ± 0.10		1.45 ± 0.15		1.48 ± 0.22		0.73 ± 0.07	
< 55	11	1.03 ± 0.02		0.69 ± 0.14		1.53 ± 0.09		1.60 ± 0.31		0.76 ± 0.07	
Tumor size			0.003		0.015		0.004		0.016		< 0.001
< 5 cm	12	1.04 ± 0.01		0.67 ± 0.12		1.56 ± 0.12		1.67 ± 0.31		0.70 ± 0.03	
≥ 5 cm	12	1.01 ± 0.02		0.78 ± 0.09		1.42 ± 0.10		1.40 ± 0.14		0.79 ± 0.06	
TNM			< 0.05		< 0.05		< 0.05		< 0.01		< 0.01
I	4	1.05 ± 0.01		0.60 ± 0.09		1.62 ± 0.14		1.89 ± 0.10		0.67 ± 0.03	
II	6	1.03 ± 0.02		0.68 ± 0.09		1.56 ± 0.11		1.70 ± 0.31		0.70 ± 0.01	
III	6	1.02 ± 0.02		0.75 ± 0.14		1.46 ± 0.06		1.41 ± 0.07		0.74 ± 0.03	
IV	8	1.01 ± 0.02		0.81 ± 0.07		1.40 ± 0.12		1.33 ± 0.05		0.81 ± 0.05	

### Overview of GO and KEGG pathway analysis

**Fig. 6:**
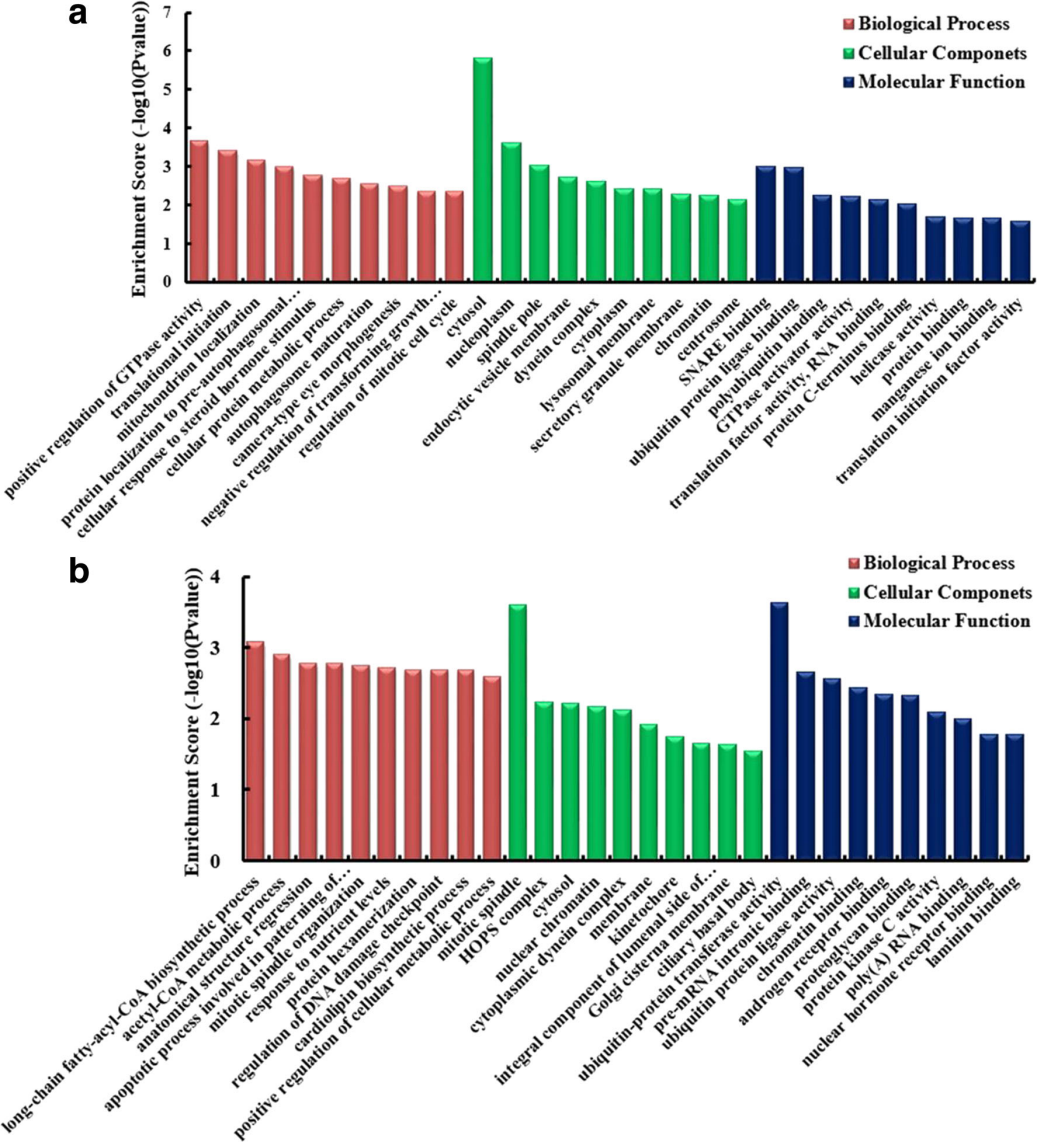
Gene Ontology (GO) analysis of the genes producing differentially expressed circRNAs. **a** GO annotation of linear counterparts of upregulated circRNAs including biological process, cellular component, and molecular function. **b** GO annotation of linear counterparts of downregulated circRNAs including biological process, cellular component and molecular function. Both up- and downregulated circRNAs are significantly changed with fold change ≥ 2, *P* < 0.05, and FDR < 0.05

Gene Ontology (GO) analysis of the genes producing differentially expressed circRNAs was used to survey whether circRNAs modulates parental gene transcription. Compared with adjacent normal tissues, the data displayed that the gene expression profile of linear counterparts of upregulated circRNAs in human CRC tissues preferred positive regulation of GTPase activity, cellular protein metabolic process, and protein binding (Fig. 6a), while GO enrichment analysis of linear counterparts of downregulated circRNAs of CRC showed that closely related GO terms were positive regulation of cellular metabolic process, acetyl-CoA metabolic process, and protein kinase C activity (Fig. 6b).

**Fig. 7:**
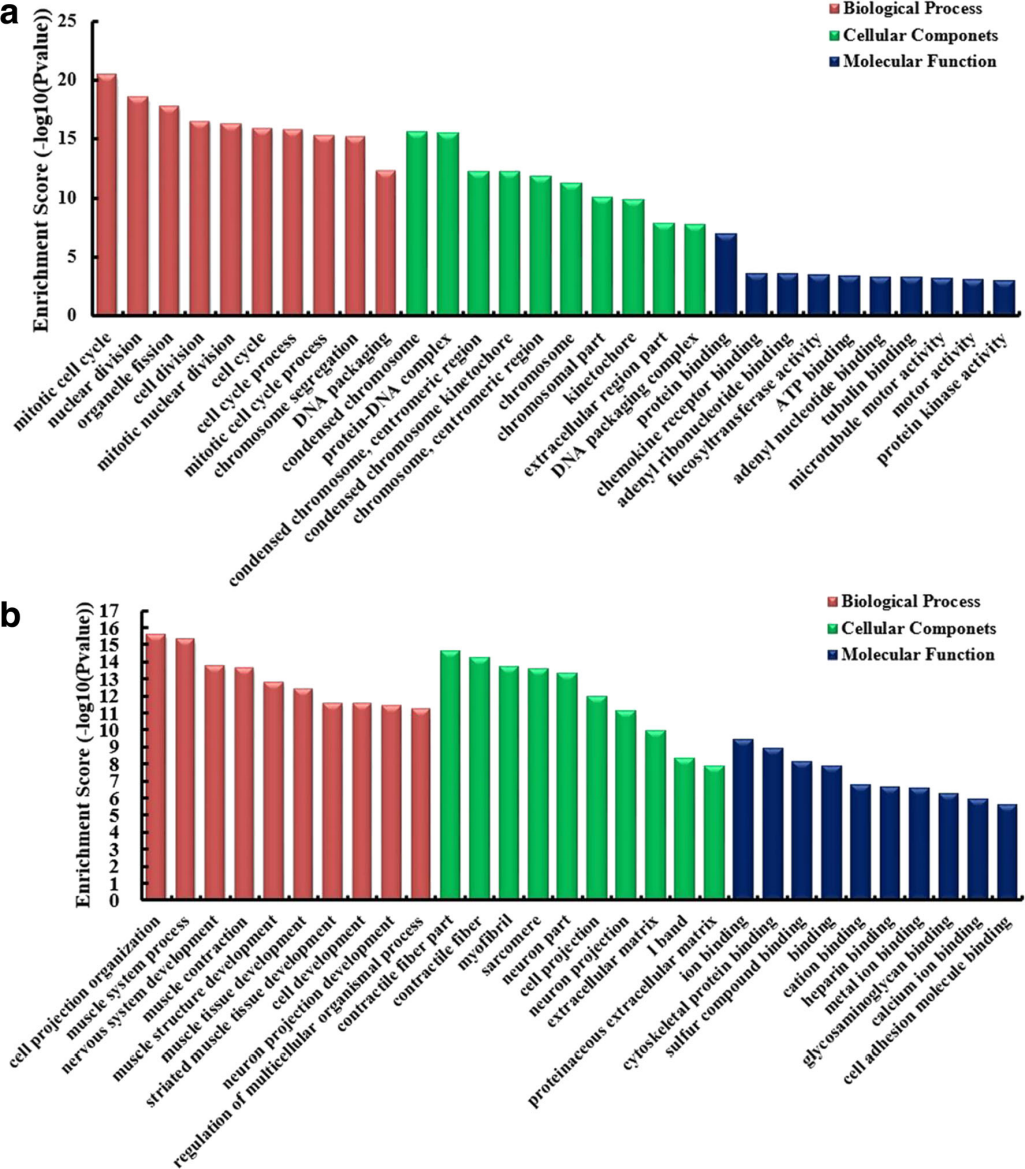
Gene Ontology (GO) enrichment analysis of lncRNA–target genes. **a** GO analysis of upregulated lncRNAs including biological process, cellular component, and molecular function. **b** GO analysis of downregulated lncRNAs including biological process, cellular component, and molecular function

**Fig. 8:**
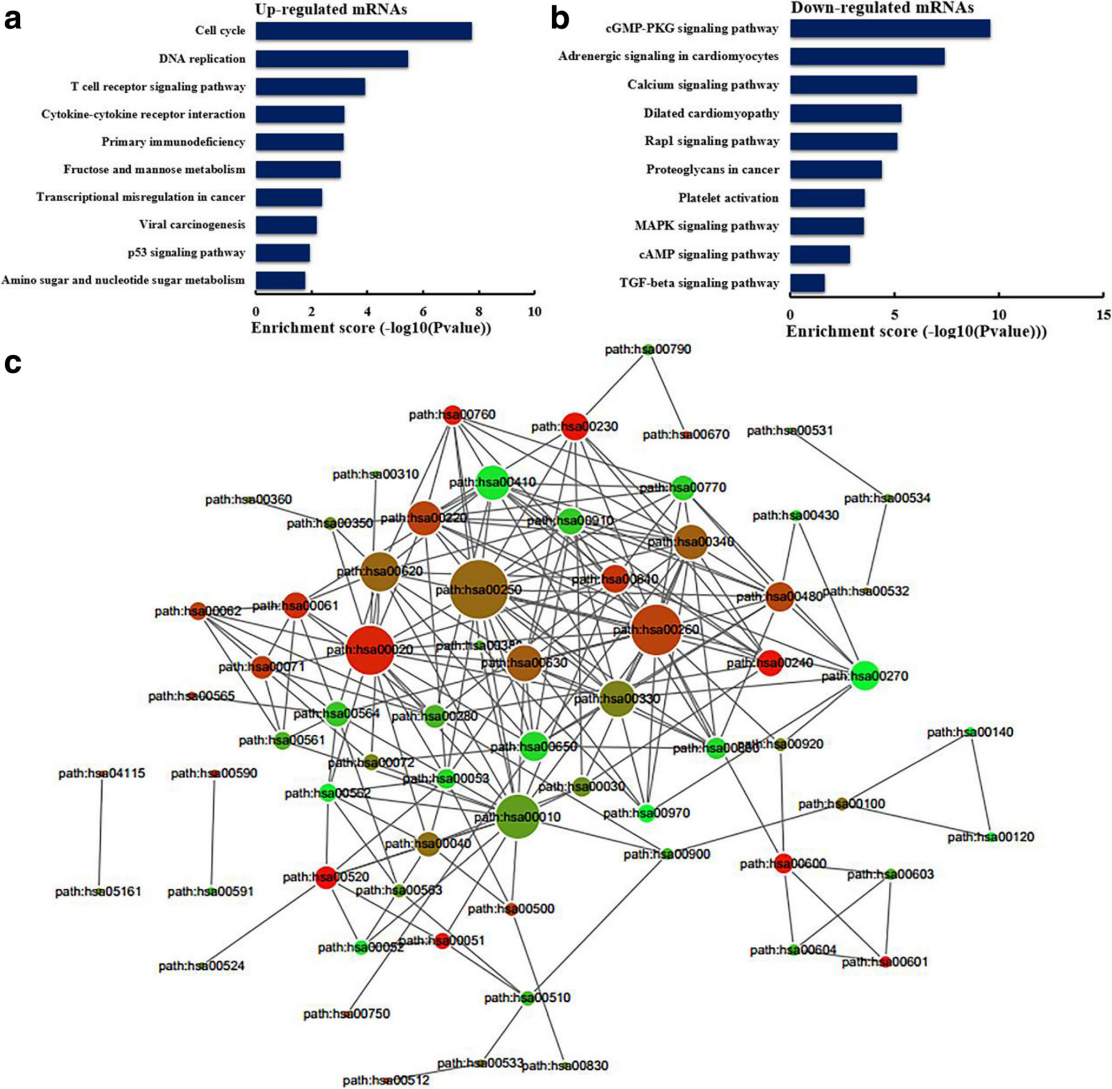
KEGG pathway enrichment analysis of lncRNA–target genes. **a** The top 10 enrichment scores in the pathway analysis of the upregulated mRNAs. **b** The top 10 enrichment scores in the pathway analysis of the downregulated mRNAs. **c** The link among pathways. Path: hsa number was Term_ID of pathway

It has been reported that lncRNAs could regulate the neighboring and overlapping coding gene expression. So, lncRNAs play their role that might be reflected on related mRNA genes. GO enrichment analysis of significant differentially expressed mRNAs can display the function of obvious differentially expressed lncRNAs. The results showed that the upregulated mRNAs, related to biological processes, were mitotic cell cycle, nuclear division, organelle fission, and cell division (Fig. 7a). Meanwhile, the downregulated mRNAs were most relevant to cell projection organization (Fig. 7b). KEGG pathway enrichment analysis for differentially expressed mRNAs was performed to understand pathways and molecular interaction related to genes. The results showed that top 10 pathways associated to upregulated mRNAs and top 10 related to downregulated mRNAs according to enrichment score. Among these pathways, T cell receptor signaling pathway and p53 signaling pathway were two important pathways in upregulated protein-coding genes, whereas cGMP–PKG signaling pathway was the top enriched KEGG pathway for downregulated transcripts (Fig. 8a, b). To explore the association between pathways, the networks were established. The links among pathways are shown in Fig.8c. The results showed that path: hsa00250 (namely alanine, aspartate, and glutamate metabolism) was the central pathway that associated with others pathways. These results suggest that these pathways might significantly play roles in the pathogenesis and development of CRC.

### Co-expression of lncRNAs/mRNAs and function predication

**Fig. 9:**
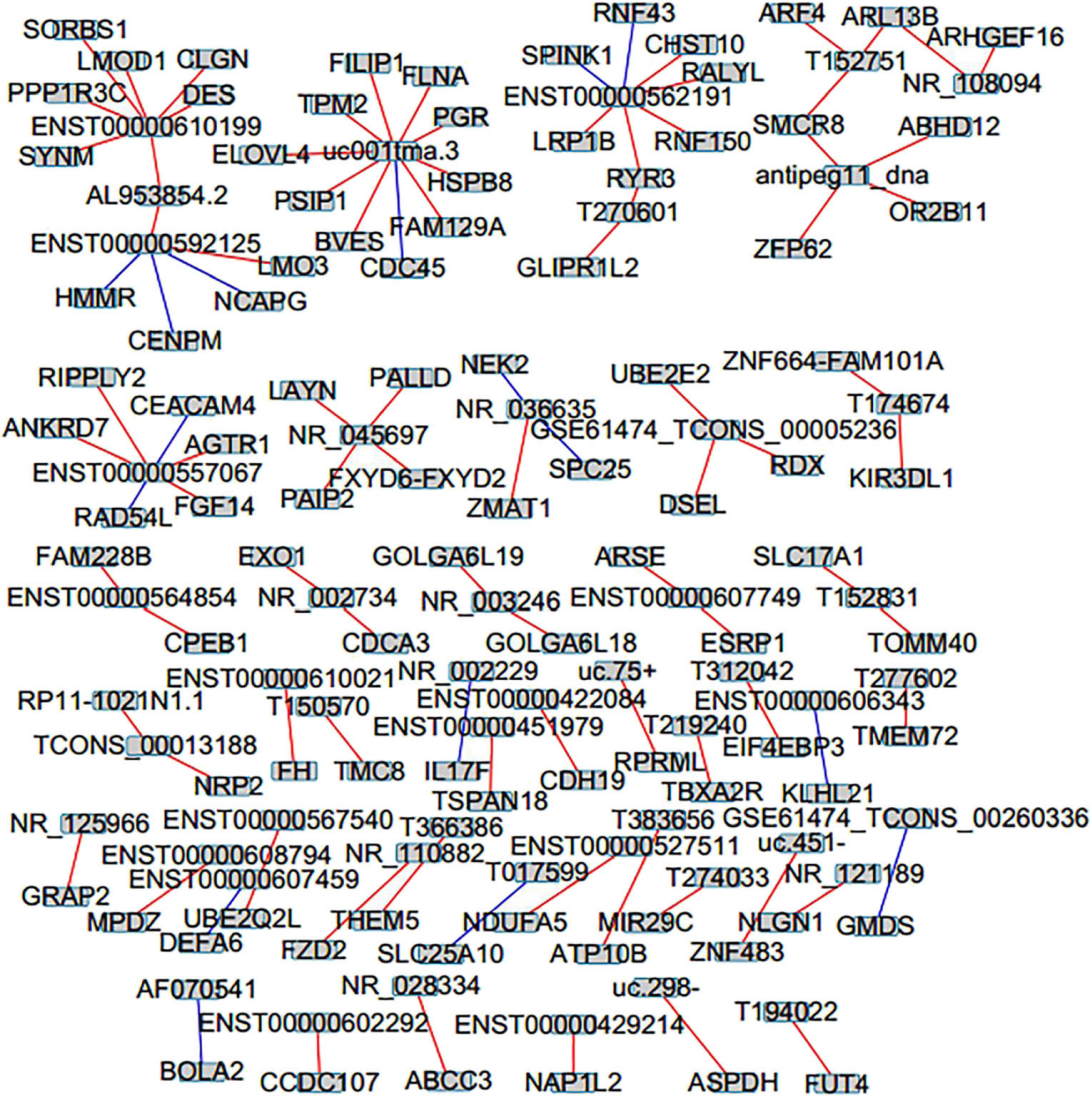
Co-expression network of mRNAs with their associated lncRNAs. The co-expression network of top 100 pairs of mRNAs and their associated lncRNAs is based on Pearson correlation coefficient (the absolute value of PCC > 0.9, *P* value < 0.01, and FDR < 0.01), and red lines mean positive correlation while green lines mean negative correlation (color figure online)

To predict the function of lncRNAs, we built co-expression of lncRNAs with their associated mRNAs basing on the degree of correlation. The top 100 pairs of co-expression of lncRNAs and their associated mRNAs are shown in Fig. 9. The network revealed that certain lncRNA was associated with one, two, or more mRNAs. For example, downregulated lncRNA uc001tma.3 was negatively with CDC45 and positively with ELOVL4, BVES, FLNA, HSPB8, PSTP1, FILIP1, FAM129A, TPM2, and PGR, while upregulated lncRNA NR_110882 was positively with FZD2. These associated mRNAs are implicated in a number of biological processes, such as cell differentiation, cell cycle, and cell development. Downregulated lncRNA NR_002229 was negatively with IL17F, while upregulated lncRNA T219240 was positively with TBXA2R. The two associated mRNAs were enriched in angiogenesis. Upregulated lncRNA T152751, NR_108094, and T150570 were positively with ARF4, ARHGEF16, and TMC8, respectively. These three associated mRNAs were implicated with apoptotic process.

### Identification of TFs in CRC-responsive lncRNAs

**Fig. 10:**
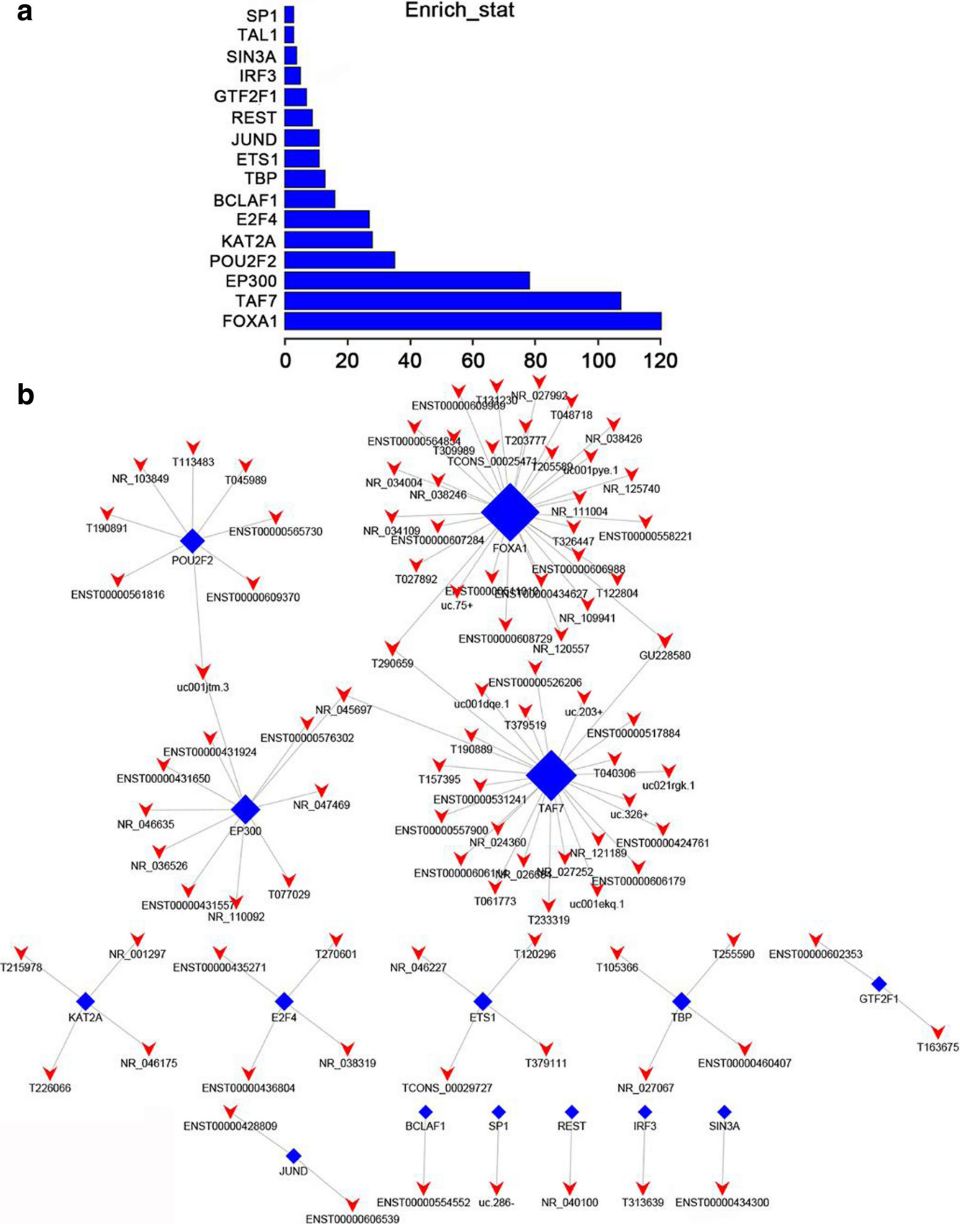
The network of enrichment transcription factors and lncRNAs. **a** The top 13 important TFs connected with lncRNAs enrichment count. **b** The TF–lncRNA network consist of 13 TFs and correlated 99 lncRNAs

**Fig. 11:**
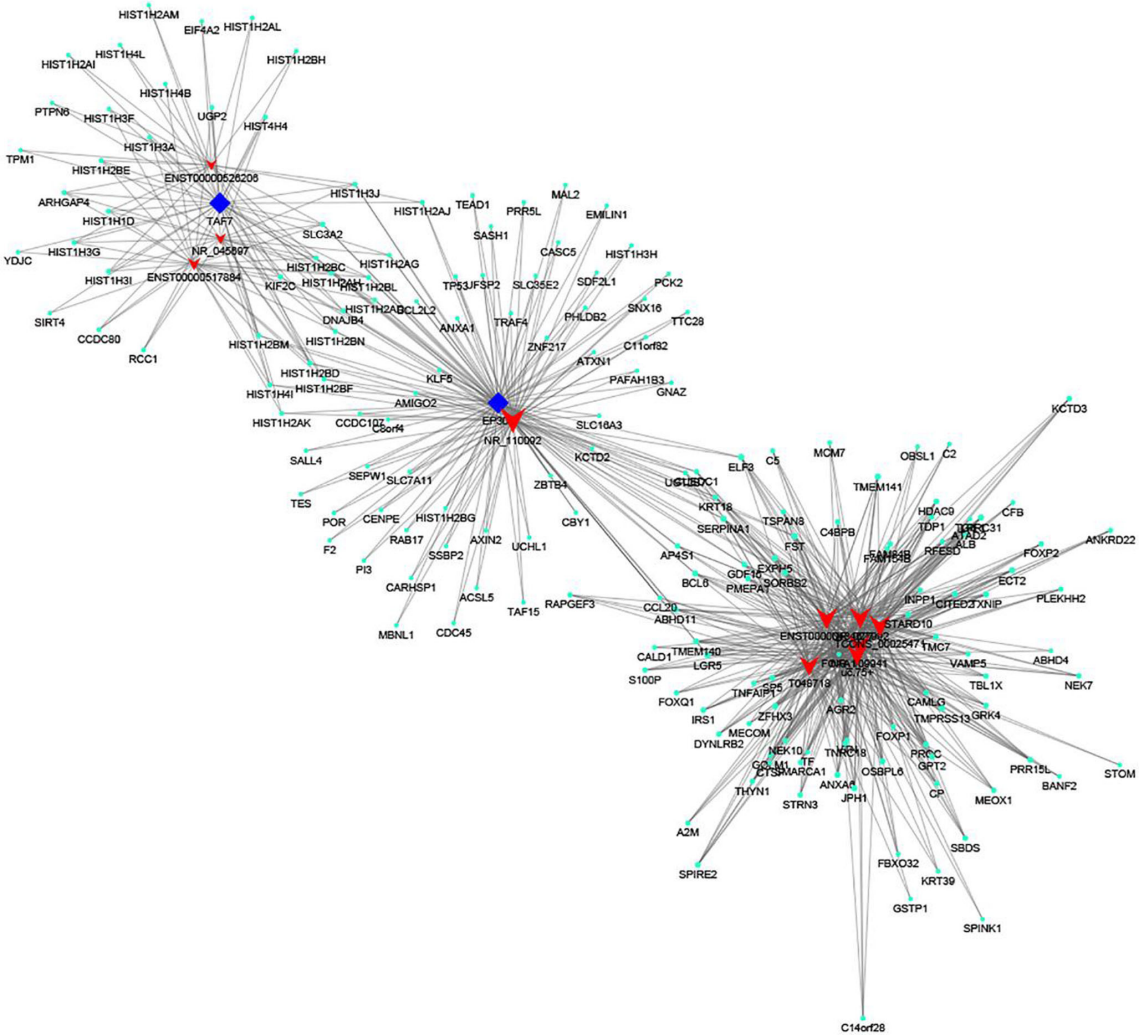
TF–lncRNA genes *trans* regulation networks of FOXA1, TAF7, and EP300

According to the enrichment with cumulative hypergeometric test, the TFs associated with lncRNAs were searched to investigate the role of lncRNAs, and then, we constructed a co-expression network of integrating differentially expressed lncRNAs with TFs. We predicted the *trans* regulatory functions of lncRNAs by the TFs that could modulate their expression. With the threshold *P* < 0.05, each TF could connect with one to more lncRNAs, and the top 13 important TFs were FOXA1, TAF7, EP300, POU2F2, KAT2A, E2F2, BCLAF1, TBP, ETS1, JUND, REST, GTF2F1, IRF3, SIN3A, TAL1, and SP1 according to enrichment count (Fig. 10a), and their correlations with lncRNAs are shown in Fig. 10b, respectively. Furthermore, we also constructed the TF–lncRNA–target gene networks of top 3 TFs, namely FOXA1, TAF7, and EP300 (Fig. 11).

## Discussion

Colorectal cancer is a vital public health problem in the world (Jemal et al. 2009). To date, about half of the patients diagnosed with this common malignancy will recur and die of disease within 5 years of diagnosis. The 5-year survival rate of CRC patient is still low (Edwards et al. 2010). Therefore, it is important for investigating the molecular mechanism in CRC. Before the discovery of many non-coding RNAs including lncRNAs and circRNAs, the conventional study of gene regulation concentrated on protein-coding genes. But, at present, some researchers found lncRNA expression was dysregulated in various cancers which suggest that abnormal lncRNA expression might play role in tumorigenesis and progression (Modali et al. 2015; Wang et al. 2015a). Lately, differentially expressed circRNAs also show the potential roles in cancer (Bachmayr-Heyda et al. 2015; Li et al. 2015a). However, comprehensive analyses of differentially expressed profiles of circRNAs and lncRNAs in CRC did not been reported. To explore the functions of circRNAs and lncRNAs in colorectal cancers, here, using microarray, we showed expression profiles of circRNAs and lncRNAs in the genome-wide for six colorectal cancer and the adjacent tissues.

By microarray, a landscape of circRNAs and lncRNAs expression was gained. Total 99, 338, and 553 upregulated and 166, 810, and 1000 downregulated circRNAs, lncRNAs, and mRNAs were verified to present significantly differential expression in CRC, respectively. About 75.85% dysregulated circRNAs belong to exonic, and 16.11% lncRNAs belong to intergenic. It has been reported that 39 circRNAs were significantly differentially expressed between normal colon mucosa and CRC samples, 11 of them upregulated in cancer and 28 downregulated (Bachmayr-Heyda et al. 2015). Circ_001569 was upregulated, while Cir-ITCH was typically downregulated in CRC in comparison with peritumoral tissue (Huang et al. 2015; Xie et al. 2016). And 762 lncRNAs (390 upregulated and 372 downregulated) were remarkably differently expressed between CRC tissues and normal tissues (Xue et al. 2015). Two thousand six hundred and thirty-six lncRNAs were differentially expressed in the CRC tissues from patients with liver metastasis, including 1600 upregulated and 1036 downregulated, compared with the CRC tissues without metastasis (> twofold), and 1584 mRNAs (548 upregulated and 1036 downregulated) were differentially expressed (Chen et al. 2016a). To our knowledge, it is first time to comprehensive report the differentially expressed circRNAs, lncRNAs, and mRNAs in CRC tissues of patients.

Subsequently, by GO analysis, we found that the gene expression profile of linear counterparts of upregulated circRNAs in human CRC tissues preferred positive regulation of GTPase activity, cellular protein metabolic process, and protein binding, while that of downregulated circRNAs of CRC preferred positive regulation of cellular metabolic process, acetyl-CoA metabolic process, and protein kinase C activity. Upregulated mRNAs, related to biological processes, were mitotic cell cycle, nuclear division, organelle fission, and cell division; meanwhile, the downregulated mRNAs were most relevant to cell projection organization. Previous study showed that GTPase, such as Kras, was involved in colorectal carcinogenesis and tumor progression (Wicki et al. 2010). Protein kinase C activation can reduce tumor growth of CRC cells (Glimelius et al. 2010), and protein kinase C beta II could inhibit CRC by modulating IGF-1-mediated cell survival (Dowling et al. 2016). And it has been reported that upregulated genes were significantly enriched in cell division in CRC tissues (Liang et al. 2016).

Then, KEGG pathway analysis showed that p53 signaling pathway was an important pathway in upregulated protein-coding genes, whereas cGMP–PKG signaling pathway was the top enriched KEGG pathway for downregulated transcripts. It has been reported that p53 signaling pathway was found to be activated in CRC (Han et al. 2014). Tumor suppressor p53 prevented cancer development via initiating cell cycle arrest, cell death, and repair of antiangiogenesis processes (Zhang et al. 2015). Ribosomal protein S15A promoted malignant transformation through misregulation of p53 signaling pathway (Chen et al. 2016b). And homeodomain interacting proteins, a serine/threonine kinase, drove p53 activation to limit CRC cell growth (Rey et al. 2013). For cGMP–PKG signaling pathway, previous study indicated that dopamine (DA) and DA type-1 receptors suppressed cell viability and invasion and induced apoptosis through cGMP–PKG signaling pathway in multiple breast cancer cell lines (Borcherding et al. 2016). And cGMP/PKG-Iα signaling pathway contributes to preventing spontaneous apoptosis and accelerating cell proliferation in both normal cells (bone marrow stromal cells and vascular smooth muscle cells) and certain cancer cells (ovarian cancer cells) (Wong et al. 2012).

Moreover, the functions of most lncRNAs are unclear until now. So lncRNA–mRNA co-expression network was constructed to estimate the function of lncRNAs (Zhao et al. 2015). LncRNA–mRNA co-expression analysis indicated that downregulated lncRNA uc001tma.3 was negatively with CDC45 and positively with elongation of very long chain fatty acids-like 4 (ELOVL4), blood vessel epicardial substance (BVES), filamin A (FLNA), heat shock protein family B (small) member 8 (HSPB8), PP2C-family Ser/Thr phosphatase 1 (PSTP1), filamin A interacting protein 1 (FILIP1), family with sequence similarity 129 member A (FAM129A), tropomyosin 2 (TPM2), and progesterone receptor (PGR), while upregulated lncRNA NR_110882 was positively with frizzled class receptor 2 (FZD2). These associated mRNAs are involved in a number of biological processes, such as cell differentiation, cell cycle, and cell development. It has been reported that TPM2 was found to be downregulated in CRC, and TPM2 loss was related with RhoA activation and tumor proliferation in CRC (Cui et al. 2016). BVES prevented epithelial-mesenchymal transition (EMT), and its epigenetic silencing may be a vital step in EMT programs during colon carcinogenesis (Williams et al. 2011). HSPB8 was implicated in the mechanisms that modulate cell cycle and cell migration in breast cancer cells (Piccolella et al. 2017). Thus, we speculated that these lncRNAs might be associated with carcinogenesis of CRC by modulating co-expression genes.

In addition, previous study indicated some lncRNAs were modulated by TFs (Wang et al. 2015b). The lncRNA–TF network analysis showed that the most relevant TFs were FOXA1, TAF7, and EP300. Previous study demonstrated that FOXA1 may be regarded as a potential prognostic marker and may facilitate tumor growth of CRC by upregulating YAP expression (Ma et al. 2016). TAF7 contributed to the transcription activation by c-Jun that regulated expression of specific target genes in various cellular processes including proliferation, stress response, and tumorigenicity (Munz et al. 2003). EP300 plays a major role in the reprogramming events, leading to a more malignant phenotype with the acquisition of drug resistance and cell plasticity, a characteristic of metaplastic breast cancer (Asaduzzaman et al. 2017). Our results suggested that lncRNAs might play role in carcinogenesis of CRC through these TFs. But the relationship between lncRNAs–TFs needs to be further surveyed.

In conclusion, here, we verified a series of differentially expressed circRNAs, lncRNAs, and mRNAs in CRC patients. The potential roles of circRNAs, lncRNAs, and mRNAs were predicted through bioinformatics analyses. These differentially expressed circRNAs, lncRNAs, and mRNAs might provide new targets for studying the molecular mechanism of colorectal cancer and lay the foundation for further study on the potential roles of circRNAs and lncRNAs in colorectal cancer.

## Electronic supplementary material

Figure S1 A-E Relative expression levels of one circRNA, one lncRNA and three mRNAs are shown comparing blood of CRC patients (A) and healthy control (N). (PNG 112 kb)

High Resolution Image (TIF 7.87 mb)

ESM 2 (XLSX 134 kb)

ESM 3 (XLSX 537 kb)

ESM 4 (XLSX 880 kb)

ESM 5 (XLSX 9.80 kb)

## Abbreviations

CRC: colorectal cancer
LncRNAs: long non-coding RNAs
CircRNAs: circular RNAs
qRT-PCR: real-time quantitative PCR
GO: Gene Ontology
KEGG: Kyoto Encyclopedia of Genes and Genomes
DE: differentially expressed
PCC: Pearson correlation coefficient
A: tumor tissues
N: adjacent normal tissues.
