# Supplementary figures and images for: Comprehensive analysis of microarray expression profiles of circRNAs and lncRNAs with associated co-expression networks in human colorectal cancer
Source: Funct Integr Genomics. 2018 Nov 16;19(2):311–27. doi: 10.1007/s10142-018-0641-9 (PMC6394731; doi:10.1007/s10142-018-0641-9)

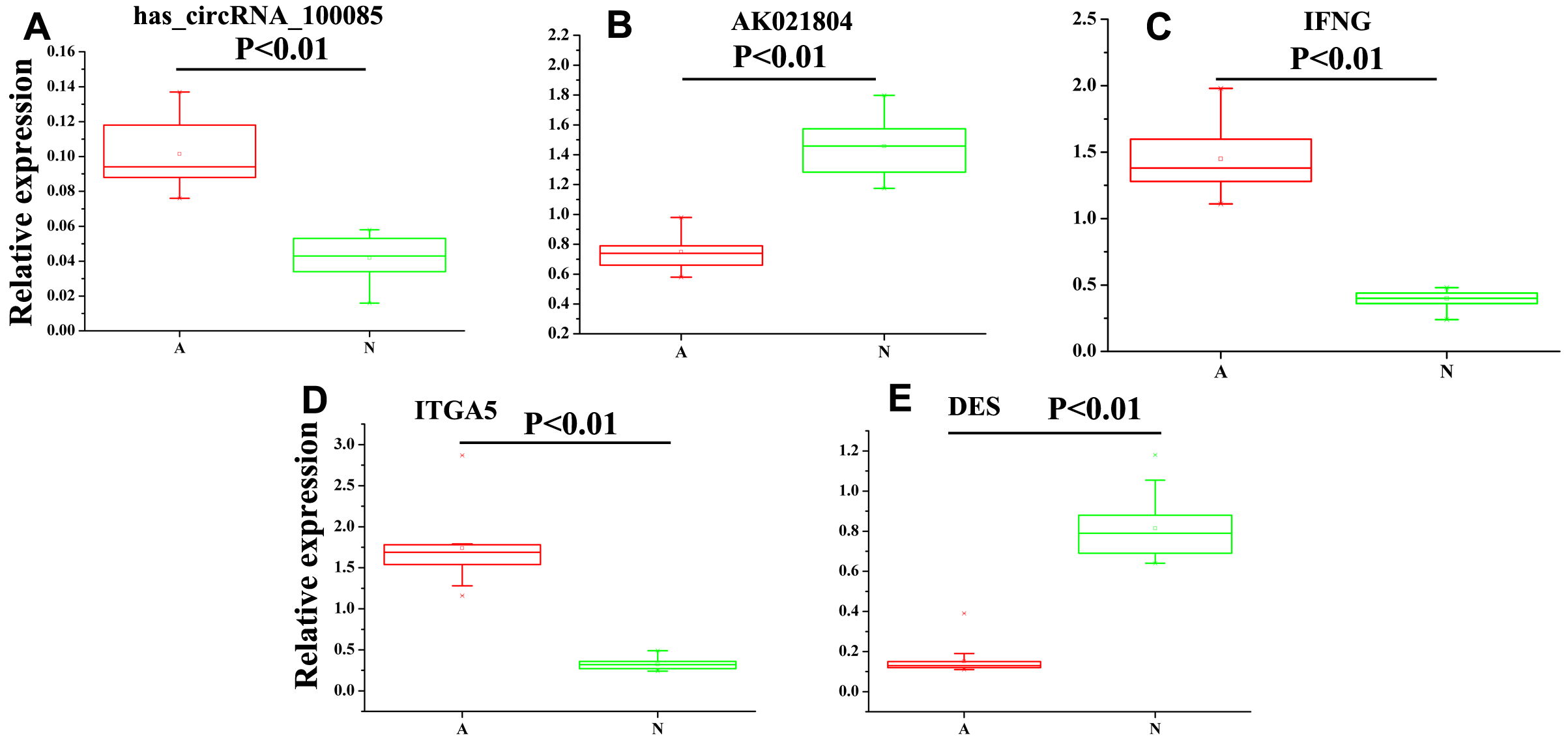

Supplement: Supplementary file 1 — A-E Relative expression levels of one circRNA, one lncRNA and three mRNAs are shown comparing blood of CRC patients (A) and healthy control (N). (PNG 112 kb) [file 10142_2018_641_Fig12_ESM.png]

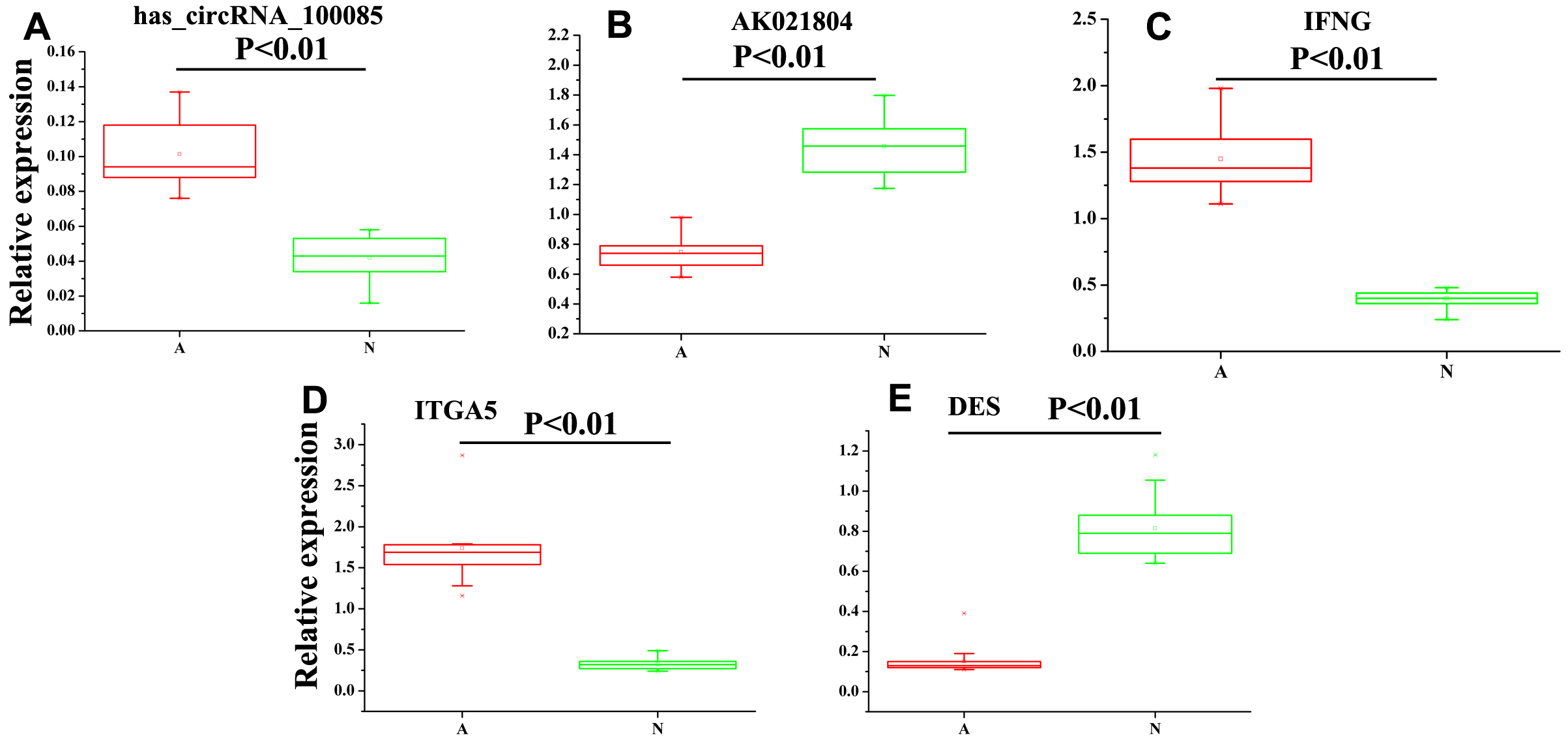

Supplement: Supplementary file 2 — High Resolution Image (TIF 7.87 mb) [file 10142_2018_641_MOESM1_ESM.tif]
